# Pilomatrix carcinoma presenting as an extra axial mass: clinicopathological features

**DOI:** 10.1186/1746-1596-3-47

**Published:** 2008-11-29

**Authors:** Noel J Aherne, David A Fitzpatrick, David Gibbons, John G Armstrong

**Affiliations:** 1 Department of Radiation Oncology, St. Luke's Hospital, Dublin, Ireland; 2 Department of Pathology, St. Luke's Hospital, Dublin, Ireland

## Abstract

Pilomatrix carcinoma is the rare malignant counterpart of pilomatrixoma, a skin adnexal tumour originating from hair matrix cells. Pilomatrix carcinoma can arise as a solitary lesion *de novo*, or through transformation of a pilomatrixoma. Pilomatrixoma was first described erroneously as being of sebaceous gland origin but was later discovered to be derived from hair matrix cells. They are rare, slow growing tumours of the skin found in the lower dermis and subcutaneous fat and are predominantly found in the neck and the scalp. While known to be locally aggressive, no malignant form was thought to exist until it was described relatively recently. Since then, approximately ninety cases of pilomatrix carcinoma have been reported.

We report the case of a 41 year old mentally retarded male who had a longstanding lesion in the left neck for approximately fifteen years previously diagnosed as a pilomatrixoma. He presented with severe headache, falls and visual disturbance and a biopsy showed pilomatrix carcinoma of the occipital region which, on computed tomography ( CT ) invaded the occipital bone, the cerebellum and the left temporal lobe. At his initial presentation he had a craniotomy and subtotal excision of the lesion but received no adjuvant therapy. After an early intracranial recurrence he had further debulking and adjuvant external beam radiotherapy. He has had no further intracranial recurrence after three and a half years of follow-up. Here we present the pathological features of this uncommon tumour.

## Background

Pilomatrix carcinomas are the aggressive variant of pilomatrixomas, a type of hair matrix tumour. Although initially thought to be benign when first described by Malherbe and Chenantais [1], as early as 1927 Gromiko [2] noted that some tumours had biologically aggressive behaviour. In 1980, Lopansri and Mihm [3] reviewed six cases of biologically aggressive pilomatrixoma. They found that the major discriminators of malignancy when compared to pilomatrixomas were the presence of hyperchromatic, vesicular basaloid cells with numerous mitoses and infiltration into adjacent tissue or blood vessels. They are characterised by sheets and islands of proliferating atypical basaloid cells with an infiltrating border. This case demonstrates an unusual pattern of local spread into the cranial cavity.

## Case presentation

A 41 year old male presented with a cutaneous tumour in the left posterior triangle of the neck which had been present for at least fifteen years. He had a history of mild mental retardation, bilateral undescended testes and had undergone appendicectomy as a child. He was otherwise well and in fulltime employment. He had multiple prior resections of the neck lesion which revealed benign pilomatrixoma. The recurrent mass measured 4.5 centimetres and was resected with clear margins. Pathologic analysis revealed a pilomatrix carcinoma. The patient had no further treatment at that time.

**Figure 1:**
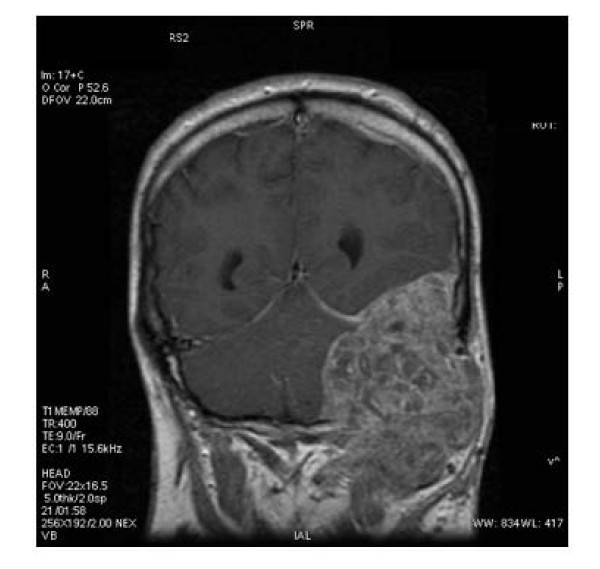
**MR features of intracranial pilomatrix carcinoma**. Coronal T1 weighted image (post intravenous contrast) demonstrating invasion of skull base with extension into intracranial cavity on left side from a large volume enhancing tumour mass arising from the cutaneous surface.

He represented 7 months later with a two week history of falls associated with severe occipital headache, visual disturbance and a left sided neck mass. Prior to neurosurgical evaluation a Magnetic Resonance (MR) scan was performed (Figure 1). This showed a 5.5 × 4 × 5 centimetre mass arising from the extra-axial tissues. This mass had a large intra-cranial component causing significant compression of the left side of the cerebellum with some mass effect. There was associated midline shift and thrombosis of the right transverse sinus. The patient proceeded to posterior fossa exploration and subtotal resection.

On postoperative MR imaging of the area an early recurrence was identified at three months, necessitating further subtotal debulking. He was then referred for adjuvant radiation therapy. The patient received fractionated three dimensional external beam radiation therapy to the occiput and left neck to a total dose of 50 Gy in 25 fractions. Serial follow-up MR scans have shown no progression in size of the intracranial component and the patient remains asymptomatic after 42 months follow-up.

**Figure 2:**
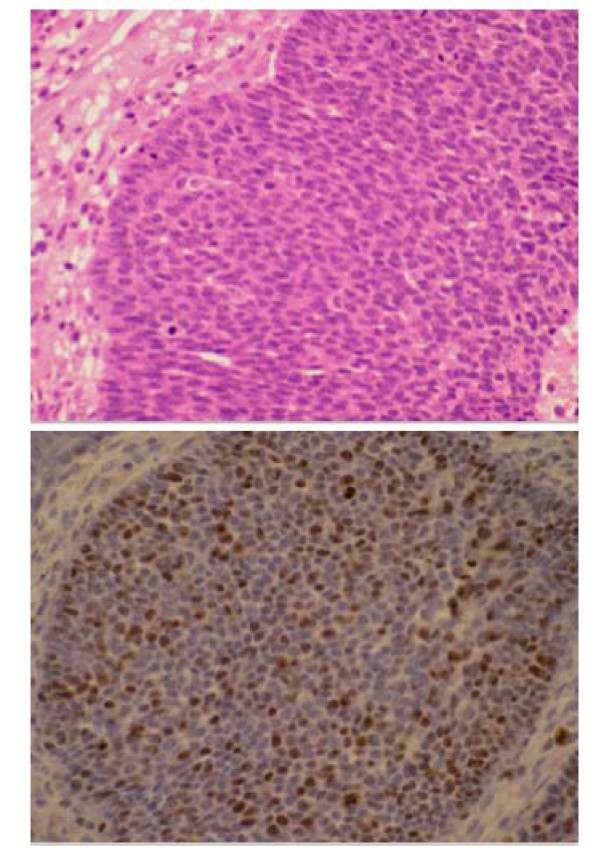
**Pilomatrix carcinoma**. The top panel (Fig. 2a) shows atypical basaloid epithelium with mitotic activity (H&E, × 400). The bottom panel (Fig. 2b) demonstrates high proliferative rate of basaloid epithelium on an MIB1 immunohistochemical stain (× 400).

In our case, haematoxylin and eosin stained sections (Figure 2a) of the recurrent tumour mass show characteristic features of pilomatrixoma. These include both basaloid and squamous epithelium with abrupt keratinization giving the characteristic 'ghost' cells. A loose fibrovascular stroma is seen and dystrophic calcification and ossification are also present. The basaloid element is hyperchromatic and in areas shows a brisk mitotic rate, including abnormal forms. An immunohistochemical stain for the proliferation marker Ki-67 (MIB1) (Figure 2b) shows the basaloid cells to be actively proliferating with more than 50% of nuclei positive in areas. No vascular space invasion is seen. These histologic features, supported by the immunohistochemistry and the clinical course allow a diagnosis of pilomatrix carcinoma to be made. In published series, the most reliable indicators of malignancy in pilomatrixomas are atypical and frequent mitoses, nuclear pleomorphism, central necrosis, ulceration and infiltration of skin, soft tissue, blood vessels or lymphatics [4].

## Discussion

Since Lopansri and Mihm first noted the existence of pilomatrix carcinomas in 1980 there have been fewer than ninety cases descibed and there has been only one previously reported case of invasion of the cranial vault [5]. In the decades since Gromiko [2] noted the aggressive behaviour of some pilomatrixomas they had been thought to follow an indolent course with little or no potential for metastasis. The morphological features of pilomatrix carcinomas appear to be nuclear pleomorphism, frequent and atypical mitoses, central necrosis, infiltration of skin and soft tissues, blood and lymphatic vessel infiltration, and ulceration [4,6,7]. The major diagnostic dilemma is in differentiating pilomatrix carcinoma from the more common pilomatrixoma and certain forms of basaloid lesions, such as adenoid basal lesions found in the cervix uteri. These adenoid basal lesions show peripheral palisading, but without any stromal reaction and have no malignant potential [8,9]. In the largest published series of pilomatrix carcinomas to date, Sau [10] et al. have demonstrated that pilomatrix carcinomas have a predilection for males and are commonly located on the head and neck. In their series of twenty patients, at least one died of metastatic disease.

Fayyazi and colleagues have assessed expression of the cell proliferation associated antigen Ki 67 (MIB1) in 15 pilomatrixomas [11]. Histologically, four epthelial components within pilomatrixomas were distinguished, representing separate stages of tumour cell differentiation. The expression of Ki-67 was highest in the basal basophilic cells of the epithelial compartments (> 70% cells) and was absent in the mesenchymal components. The expression of Ki-67 in greater than 50% of the basaloid cells in our case, in combination with the local aggressiveness and the multiple recurrences makes the diagnosis of pilomatrix carcinoma.

## Conclusion

Pilomatrix carcinomas are locally aggressive tumours which have a propensity for recurrence, especially when incompletely excised. Wide excision is the preferred treatment and radiation therapy should be considered in the presence of adverse features such as recurrent disease or residual macroscopic disease, as in our case. In the cases where adjuvant chemotherapy and radiation have been used there are instances of apparent cure with these as well as cases with progression and death, despite treatment. In recurrent pilomatrix carcinoma, no chemotherapy regimen has been demonstrated to provide local control or to halt metastatic spread. The diagnosis should always be considered in the differential of any recurrent skin lesion with locally invasive behaviour.

## Consent

Written informed consent was obtained from the guardians of the patient for publication of this Case Report and the accompanying images. A copy of the written consent is available for review by the Editor-in-Chief of this journal.

## Competing interests

The authors declare that they have no competing interests.

## Authors' contributions

NA drafted and revised the manuscript, as well as completing the literature search. DF supplied the clinical information on the patient, and critically evaluated the manuscript. DG prepared all the pathological material and revised the manuscript. JA devised the concept for the manuscript. All authors have read and approved the final manuscript.
